# Two‐Dimensional Alloying at the Iron‐Copper Interface in Steel Driven by Magnetic Friedel Oscillations

**DOI:** 10.1002/advs.202514699

**Published:** 2025-10-30

**Authors:** Wen‐Qiang Xie, Jin‐Li Cao, Jian‐Long Kou, Wen Tong Geng

**Affiliations:** ^1^ Department of Physics Zhejiang Normal University Jinhua 321004 China; ^2^ School of Materials Science and Engineering Hainan University Haikou 570228 China; ^3^ Institute of Reactor Engineering and Technology China Institute of Atomic Energy Beijing 102413 China

**Keywords:** density functional theory, iron‐copper interface, magnetic Friedel oscillations, nanoscale copper precipitates, 2D alloying

## Abstract

Copper precipitation is widely used in steel engineering, but the nanoscale size of early‐stage Cu precipitates makes determining their composition experimentally challenging. The late Professor Morris Fine highlighted the puzzling discrepancy between the low solubility of Fe in bulk Cu and the surprisingly high Fe content in Cu precipitates revealed by atom probe tomography. Using rigorous first‐principles density functional theory calculations, stable Fe/Cu interfacial structures are systematically searched for and find a single diffuse layer at the (001) interface, up to two diffuse layers in the [111] direction, and no diffuse atomic layer in the [110] orientation. Magnetic Friedel oscillations drive 2D alloying at the (001) interface, while configurational entropy governs alloying at (111). These interfacial alloying effects explain the significant Fe content observed in nanoscale Cu precipitates. Further calculations show that this alloying substantially alters impurity segregation at the (001) interface. In particular, enhanced hydrogen trapping at the interface suggests that Cu precipitates may help mitigate hydrogen embrittlement in steel. These findings reveal how quantum interference effects can drive interfacial mixing and open new avenues for atomic‐scale alloy design guided by electronic structure.

## Graphical abstract

The interlayer magnetic couplings (left) and the changes of atomic‐layer‐averaged electron density (right) for Fe and Cu sides near the sharp and diffuse Fe/Cu (001) interfaces reveal that magnetic Friedel oscillations at Fe/Cu interfaces in steel drive atomic‐scale 2D alloying, resolving the long‐standing paradox of high Fe content in Cu precipitates.

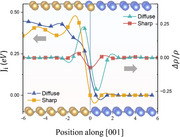

## 1 Introduction

The strengthening effect of Cu precipitates in steels is primarily attributed to their ability to impede dislocation motion, enhancing the material's mechanical properties, especially in high‐strength low‐alloy steels and weathering steels. During aging, fine, nanoscale copper precipitates form in the copper‐containing steel matrix, and they act as obstacles to dislocation movement, thereby increasing the yield strength and hardness of steels. The strengthening impact is particularly effective when the precipitates are coherent with the matrix, as they can strongly interact with dislocations.^[^ ^1^ ^]^ One of the recent advances in the study of copper precipitation strengthening in steels has focused on improving the understanding of nanoscale mechanisms, which is indispensable in enhancing processing methods and expanding the applications of Cu‐strengthened steels.^[^ ^2^, ^3^ ^]^ On the other hand, Cu precipitates have also been found to improve the steels’ resistance to hydrogen embrittlement,^[^ ^4^ ^]^ suggesting that they can serve as hydrogen traps.

Due to the minuscule size of Cu precipitates, often just a few nanometers in diameter, and its excellent lattice match with the Fe matrix (See Figure S1, Supporting Information for a schematic diagram highlighting the interfacial coherency), determining their precise composition has been a persistent challenge for experimental methodologies. This challenge was notably addressed by the late Professor Morris Fine, who highlighted a significant mystery:^[^ ^5^ ^]^ the remarkable content of Fe in Cu precipitates, as revealed by atom probe tomography (APT),^[^ ^6^, ^7^ ^]^ stands in stark contrast to the low solubility of Fe in bulk Cu.^[^ ^8^ ^]^ The APT is widely regarded as a powerful tool for analyzing the composition of nanoscale precipitates in alloys, but its accuracy depends on several factors. While it provides atomic‐scale spatial resolution and high sensitivity for elemental analysis, some limitations and challenges remain. For instance, APT detectors typically capture only 30–80% of the evaporated ions. While correction algorithms exist, they may not fully compensate for compositional errors.^[^ ^9^, ^10^, ^11^, ^12^ ^]^

The mystery about the discrepancy between APT measurements, which claim a diffuse Fe/Cu interface, and the thermodynamic argument in favor of a sharp interface has deeply captivated us for a long time. We recall that Friedel oscillations^[^ ^13^ ^]^ of electron density dictate multilayer relaxations at metal surfaces.^[^ ^14^ ^]^ Very recently, we have demonstrated by density‐functional theory (DFT) calculations that Friedel oscillations induce hydrogen accumulation near the Σ3 (111) twin boundary in face‐centered‐cubic Fe^[^ ^15^ ^]^ and exert a sizable influence on the solute‐solute interactions in Mo.^[^ ^16^ ^]^ On the other hand, magnetism in alloy steels plays a decisive role in determining their stable crystal structures—body‐centered cubic in ferritic steels, body‐centered tetragonal in martensitic steels, and face‐centered cubic in austenitic steels—which in turn govern their mechanical performance and electronic properties.^[^ ^17^, ^18^ ^]^ In a recent work, Mitsui et al. observed magnetic Friedel oscillations at the Fe (001) surface by atomic‐layer‐resolved synchrotron radiation ^57^Fe Mössbauer spectroscopy.^[^ ^19^ ^]^ All these findings strongly indicate that the long‐range nature of Friedel oscillations may have a noticeable effect on chemical bonding in metals. This sparks the intriguing idea that the charge and spin Friedel oscillations in the vicinity of the Fe/Cu interface might also have a noticeable effect on the mixing of Fe and Cu atoms at the interface within one or a few atomic layers.

## 2 Sharp Versus Diffuse Interfaces

To make a conceptual clarification of sharp and diffuse interfaces pedantically for the convenience of discussion, here we mean strictly no diffuse atomic‐layer by sharp, slightly different from the convention that interfaces with one or two diffuse atomic layers can still be called sharp.^[^ ^20^ ^]^

### 2.1 2D Alloying Model at the Fe/Cu (001) Interface

**Figure 1:**
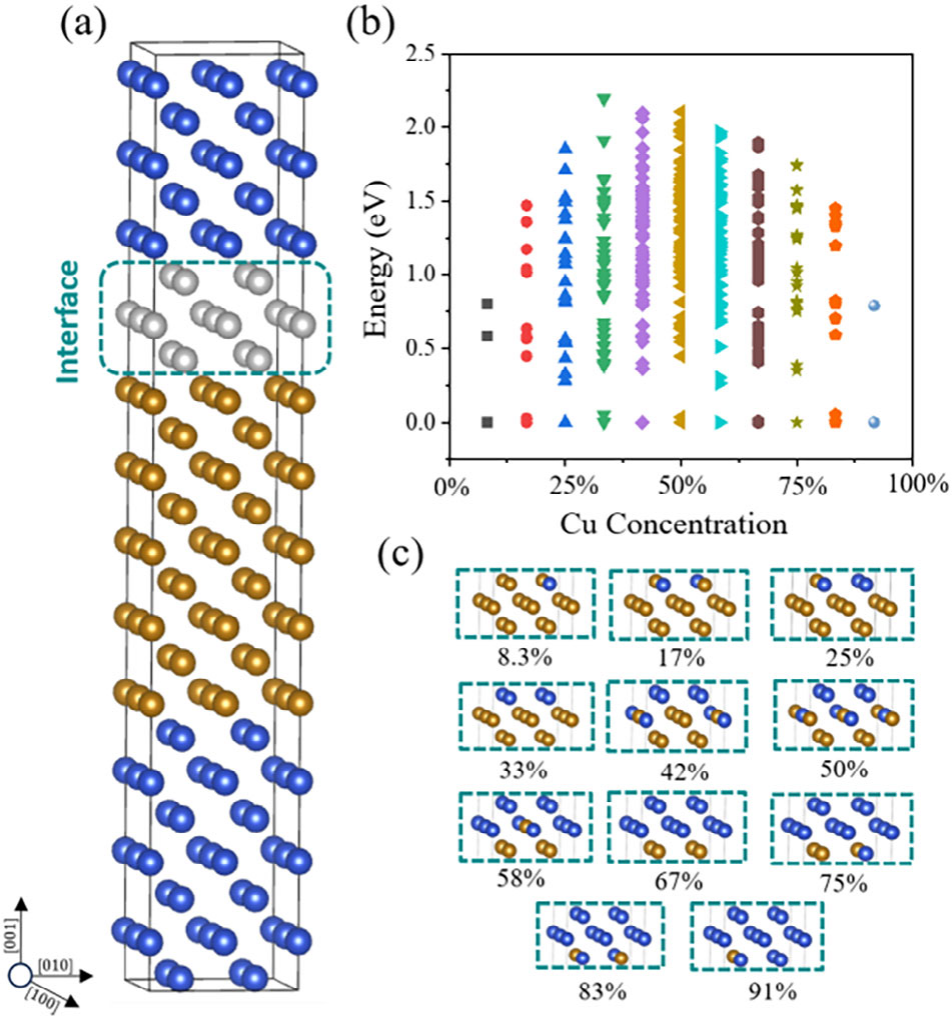
Configurations and energies at the Fe/Cu (001) interface. a) Supercell used to model the (001) interface between Fe (brown) and Cu (blue). Three‐layer gray atoms at one interface represent undesignated species, either Cu or Fe. b) The relative energy of each configuration in reference to the most stable one. c) The most stable configuration for each Cu concentration.

The observation of magnetic Friedel oscillations at the Fe (001) surface^[^ ^19^ ^]^ prompted us to focus first on the (001) precipitate/matrix interface. We have conducted an exhaustive first‐principles DFT investigation. The Fe/Cu interface was modelled using a supercell formed by stacking atomic layers, and contains two interfaces due to periodic boundary conditions. As shown in **Figure** **1**a, the supercell is 2*a* × 2*a* (*a* is the lattice constant of body‐centered‐cubic Fe) in the (001) plane and consists of 24 atomic layers in the [001] direction, and is thick enough to minimize the interaction between neighboring interfaces. Three layers (gray) at one interface between Fe (brown) and Cu (blue) were set to be variable in the composition. The content of Cu or Fe in the three mixed layers could take the value of *n*/12 (*n* = 0 to 12). Our exhaustive search has identified a total of 349 distinct structural configurations, generated using SAGAR.^[^ ^21^ ^]^ In reference to the most stable configurations, we present in Figure 1b the relative energy for each configuration of the diffuse interface for each Cu concentration. In Figure 1c, we display the most stable configuration for each Cu concentration. It can be seen that for all Cu concentrations in the three mixing‐allowed layers, the most stable Fe/Cu (001) interface is either sharp or single‐layer diffused, indicating a well‐defined boundary between the Fe and Cu, with at most a single (001) layer of Fe‐Cu mixing. To avoid confusion, we note that the Cu concentrations shown in Figure 1c correspond only to the ratio of Cu atoms in the designated three‐layer interfacial region, rather than the bulk Cu concentration in Fe.

### 2.2 The Comparative Stability of Sharp Versus Diffuse Interfaces

**Figure 2:**
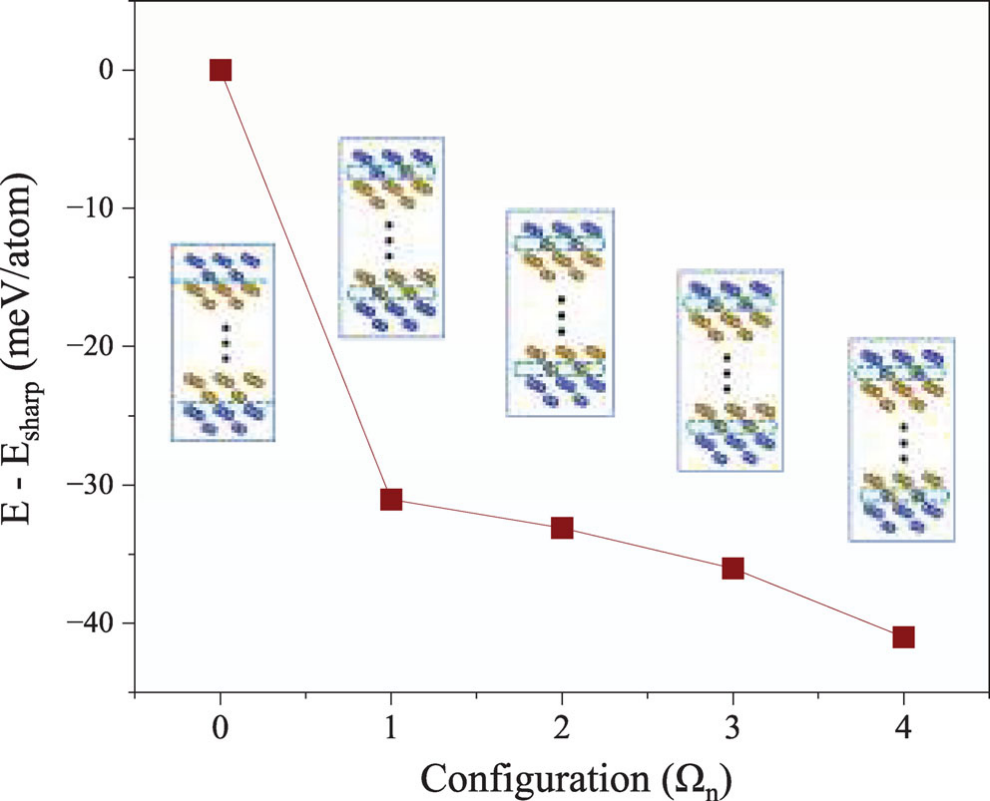
Interfacial energy of different configurations of the single‐layer diffuse Fe/Cu (001) interface in reference to the sharp interface (meV per interfacial atom). Parts of the supercells used to model the sharp (Ω_0_) and four inequivalent single‐layer diffuse (Ω_1_ − Ω_4_) interface configurations are displayed as insets.

Since the chemical potential of Cu cannot be readily determined, it is not straightforward to compare the stability of sharp and single‐layer diffuse interfaces in the above set of calculations. To overcome this difficulty, we deal with two interfaces in the supercell at the same time. We started with two sharp interfaces and designated one Fe layer at one interface and one Cu layer at the other interface, and swapped species within the two layers. There are four atoms in each layer, and there are only five inequivalent configurations, as shown in **Figure** **2**: i) one layer is purely Fe and the other purely. Cu, $\Omega_{0}$; ii) one layer has one Fe and the other has three Fe, $\Omega_{1}$; iii) both layers have two Fe and two Cu atoms, $\Omega_{2}$, $\Omega_{3}$, and $\Omega_{4}$, The configuration $\Omega_{0}$ is chosen to be the energy reference. It can be found that $\Omega_{0}$ has the highest energy, $\Omega_{1-4}$ are noticeably lower (Figure 2). The most stable configuration is $\Omega_{4}$, 0.041 eV per atom more stable than $\Omega_{0}$. Notice that including the entropy contribution makes the Gibbs free energy of the single‐layer diffuse interface even lower. These results indicate unambiguously that the diffuse interface is favorable in the (001) case. That is, 2D alloying occurs at the Fe/Cu (001) interface.

### 2.3 Charge Friedel Oscillations (CFO) and Spin Friedel Oscillations (SFO) as Driving Forces of Interlayer Binding

To understand the interfacial structure, we try to investigate the long‐range Friedel oscillations, including charge Friedel oscillations (CFO) and spin Friedel oscillations (SFO). Friedel oscillations manifest as spatial modulations in the electron density of a metal induced by a localized perturbation.^[^ ^13^ ^]^ For a planar defect, these oscillations exhibit the form:^[^ ^22^ ^]^

(1) $$\frac{\Delta \rho }{\rho }=A\frac{\cos\left(2k_{f}z+\emptyset \right)}{{\left(2k_{f}z\right)}^{2}}$$

where the magnitude *A* is mainly determined by the Fermi surface geometry, the strength of the disturbance introduced by the defect, and temperature as well, *k_f_* denotes the Fermi wave vector of the host metal's electron gas, *z* represents the distance from the defect plane, and ∅ is a phase shift attributed to electron transfer between the defect and the matrix. Such a charge density modulation can induce an observable change of interlayer distance, which indicates the variation of interlayer binding.^[^ ^15^ ^]^

To elucidate the driving force underlying the 2D alloying at the Fe/Cu (001) interface, we decompose the total energy (*E*_**Tot**_) of the supercells consisting of diffuse and sharp interfaces into the intralayer (*E*_***xy***_) and interlayer (*E_z_*) contributions,

(2) $$E_{Tot}=E_{xy}+E_{z}$$

And

(3) $$E_{z}=\sum E_{z}^{i}$$

where *i* denotes the interlayer space, which can be indexed by its separation from the interface. $E_{z}^{i}$ is defined as the cleavage energy along the *i‐th* interlayer space, i.e., the energy needed to separate the whole system into two semi‐infinite parts. In DFT calculations, it was determined by the energy increase of the whole system upon inserting a 15Å‐thick vacuum layer into the supercell at the *i‐th* interlayer space.

**Table** **1** summarizes the calculated total energy and the total interlayer binding energy of the supercell containing two sharp or two single‐layer diffuse Fe/Cu (001) interfaces, both with and without spin polarization treatment. We can see that the contribution of interlayer binding to the total energy is comparable to that of the intralayer binding, and it is the former that makes the diffuse interface more favorable. By contrast, the strong intralayer binding stabilizes the sharp interface.

**Table 1:** The interlayer binding (*E*_***z***_), intralayer binding (*E*_***xy***_), and total energy (*E*_**Tot**_) of the supercells modeling the sharp and single‐layer diffuse Fe/Cu (001) interfaces (eV).

	Spin‐polarized		Non‐spin‐polarized	
Interface	Sharp	Diffuse	Sharp	Diffuse
*E_z_*	−190.50	−192.37	−222.75	−222.13
*E_xy_*	−374.83	−374.31	−300.79	−299.11
*E*_Tot_	−565.32	−566.69	−523.54	−521.24

**Figure 3:**
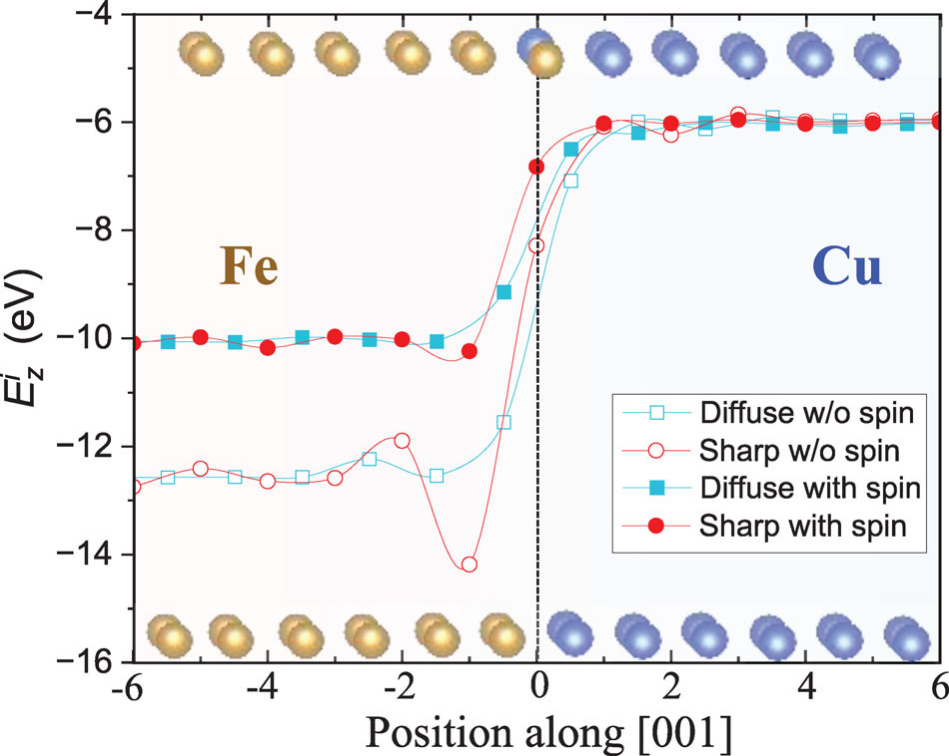
The interlayer binding energy (eV/interface) near the Fe/Cu (001) interfaces: sharp and diffuse interfaces, with and without spin‐polarization.

To examine how the interlayer binding $E_{z}^{i}$ responds to charge and spin Friedel oscillations, we display in **Figure** **3** its variation with different interlayer spaces for both sharp and mixed interfaces. In the absence of spin‐polarization, $E_{z}^{i}$ experiences a more substantial drop at the sharp interface (circles) than at the diffuse interface (squares). This is because the electronic density and spin density (Figures S2 and S3, Supporting Information) along the [001] direction undergo a more abrupt change at the sharp interface, and hence more pronounced Friedel oscillations in the interlayer charge density, which render a more significant modulation in the interlayer binding energy. The strengthening of $E_{z}^{i}$ at the interface enhances the stability of the sharp interface. Interestingly, spin‐polarization diminishes seriously the magnitude of the oscillations of $E_{z}^{i}$ in both sharp (solid circles) and diffuse (solid squares) cases, strongly suggesting the spin Friedel oscillations cancel out to some extent the effect of charge Friedel oscillations. A closer look can tell that in the vicinity of the interface, the line connecting solid circles lies lying averagely above that connecting solid squares, because of the difference in the wavelength of the oscillations. And consequently, the diffuse interface is more energetically favorable. For completeness, we also examined the interlayer binding energy for the Ω_1_ configuration and compared it with Ω_0_ (sharp) and Ω_4_ (diffuse), as shown in Figure S4 (Supporting Information). The results reveal that the oscillation behavior of Ω_1_ lies between those of Ω_0_ and Ω_4_, indicating that our main conclusions regarding Friedel oscillations and impurity segregation are robust and do not qualitatively change with Cu concentration in the diffuse Fe/Cu (001) interlayer.

To understand the effect of spin polarization, we have evaluated the isotropic magnetic interaction within the Heisenberg model^[^ ^23^ ^]^ using OpenMX^[^ ^24^ ^]^ and TB2J^[^ ^25^ ^]^ codes. OpenMX is a DFT‐based code, while TB2J is a post‐processing code based on Green's function.^[^ ^26^ ^]^ In our supercells, the magnetic coupling at the *i*_th_ interlayer space was represented by the integrated value ($J_{i}$).^[^ ^27^ ^]^ of average isotropic magnetic interaction (${J_{i}}^{\mathrm{jk}}$). The integrated value $J_{i}$ up to the given distance is calculated as:

(4) $$J_{i}=\frac{1}{2}\;\sum_{0}^{r}J_{i}^{jk}S^{2}$$

Here, A and *S* is the area of the interface in the supercell and the unit spin vector. Note that $J_{i}^{jk}$ is a function of atomic pair distance. $J_{i}$ sums up the magnetic interaction pair between atom *j* and *k*, where one atom is on one side of the 𝑖_th_ interlayer space and the other atom is on the other side of the 𝑖_th_ interlayer space. The convergence energy criterion for OpenMX was set to 10^−6^ Hartree/Bohr.

### 2.4 Oscillatory Behavior of SFO and CFO and Their Analytical Fitting

**Figure 4:**
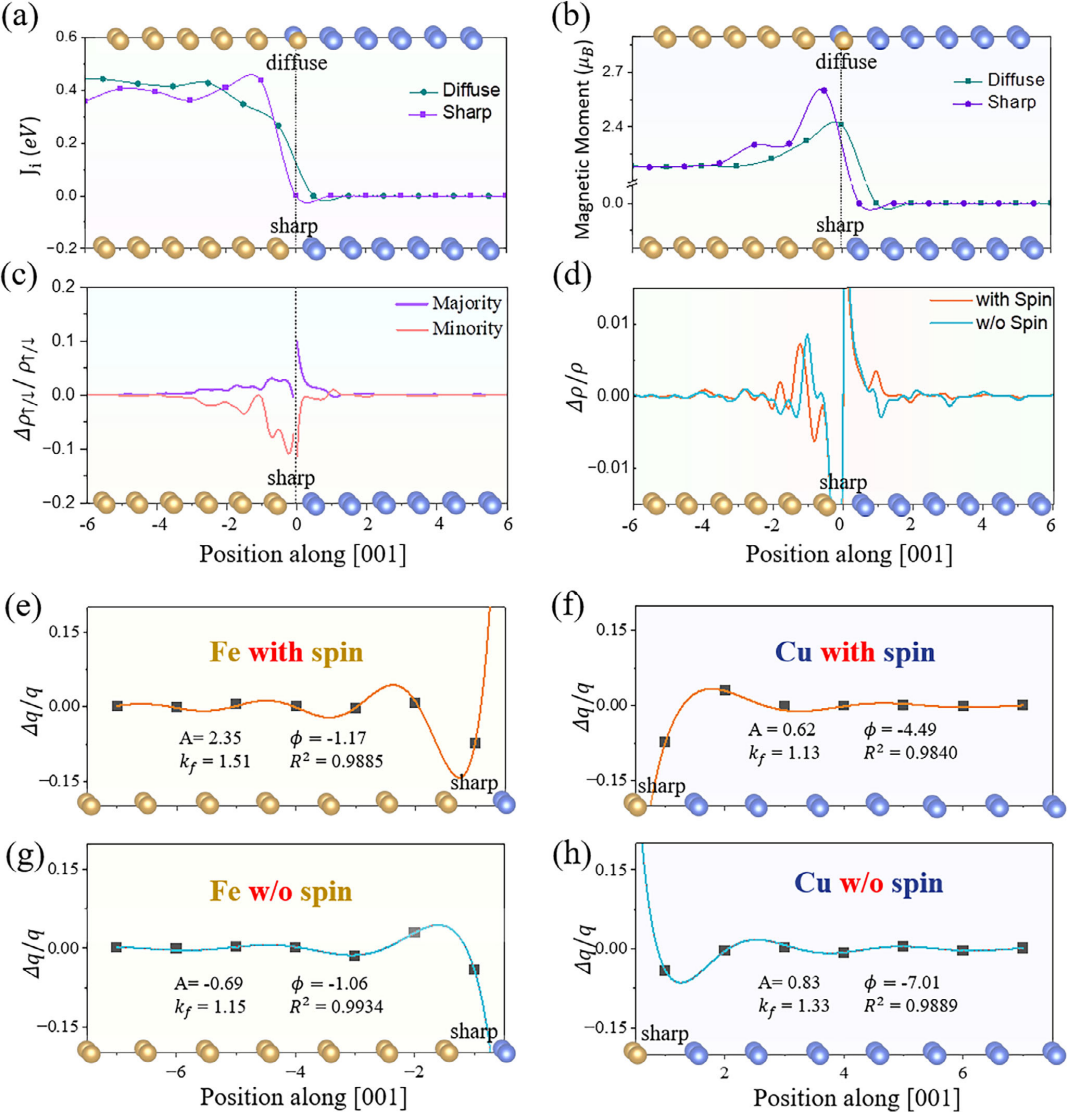
Interlayer magnetic interactions near the Fe/Cu (001) interface. a) Interlayer magnetic coupling. b) Magnetic moments. c) Change in the plane‐averaged majority and minority electron densities. d) Change in plane‐averaged electron density near the sharp interface. e–h) Change in atomic‐layer‐averaged charge near the sharp interface (black squares: DFT data; solid lines: fits to the Friedel oscillation equation [Equation (1)]), performed separately for the Fe and Cu sides, both with and without spin polarization (highlighted in red). Atomic structures near the interface are labeled as “sharp” and “diffuse” to indicate the interface position and guide the eye to the corresponding atomic layers.

Based on the magnetic interactions calculated by DFT and Green's function method (Figure S5, Supporting Information), we have studied the interlayer magnetic interaction across the (001) interface, and the results are shown in **Figure** **4**. We find in Figure 4a clear magnetic Friedel oscillations, especially for the sharp case, as was found recently at the Fe (001) surface.^[^ ^19^ ^]^ Interestingly, the oscillations in interlayer binding and interlayer magnetic interaction are in antiphase (c.f. Figure 3). To further illustrate the interplay between spin and charge Friedel oscillations, we analyzed the charge and spin density difference at the Fe/Cu (001) interface. The isosurface plots (Figure S6, Supporting Information) clearly reveal the oscillatory features and their phase relationship, while the layer‐resolved Bader charge analysis (Figure S7, Supporting Information) provides quantitative evidence of electron redistribution, which is significantly enhanced by spin polarization. Therefore, the contribution to the total energy from SFO largely cancels the contribution from CFO. Such a suppression is corroborated by the reduction in the magnetic moment of Fe near the Fe/Cu sharp interface, as is shown in Figure 4b.

Figure 4c shows the change in majority and minority electron densities upon formation of the interface, which is defined as

(5) $$\Delta \rho_{Fe}\;\left(↑/↓,z\right)=\rho_{FeCu}\left(↑/↓,z\right)-\;\rho_{Fe}\left(↑/↓,z\right)$$

in the Fe part and

(6) $$\Delta \rho_{Cu}\left(↑/↓,z\right)\;=\rho_{FeCu}\;\left(↑/↓,z\right)-\;\rho_{Cu}\left(↑/↓,z\right)$$

Clearly, the majority and minority densities vary in antiphase, with a wavelength different from that of charge density. Thus, spin polarization leads to significant electron density redistribution. To gain deeper insight into the CFO, we display in Figure 4d the difference of plane‐averaged electron density difference (**Δ**ρ/ρ) at each point, *z*, along the [001] direction between the supercell for the Fe/Cu (001) interface and one for sole Fe or Cu, with **Δ**ρ defined as:

(7) $$\Delta \rho_{Fe}\;\left(z\right)=\rho_{FeCu}\left(z\right)-\;\rho_{Fe}\left(z\right)$$

in the Fe part and

(8) $$\Delta \rho_{Cu}\;\left(z\right)=\rho_{FeCu}\left(z\right)-\;\rho_{Cu}\left(z\right)$$

in the Cu part of the supercell. We find that the wavelength of charge Friedel oscillations in Fe is slightly smaller than in Cu, and spin polarization reduces it further. Moreover, since the charge density of down spin experiences a decrease with a larger magnitude than the increase of up spin, SFO has a net compensation effect on the CFO. It is for this reason that the oscillations of interlayer binding strength are lessened in the presence of spin polarization.

To have a better perspective of the correlation between oscillations of the interlayer binding strength and those of the charge density, we integrate **Δ**ρ/ρ over each interlayer space and obtain a single value for each of them. The results are displayed in Figure 4e–h for the case with and without spin polarization. We find that the averaged change of the electron density over each interlayer region features strong Friedel‐like oscillations, like the interlayer binding strength, an indication of impressive correlation of the two. Interestingly, spin polarization switches the larger amplitude of the charge oscillation from the Fe side to the Cu side, in agreement with the cancellation between magnetic coupling and chemical bonding (Figure 3). We fit the Friedel oscillations separately in the Fe and Cu regions near the sharp (001) interface, both with and without spin polarization. We find that spin polarization increases *k_f_* in the Fe region from 1.15 to 1.51 Å^−1^ while reduces *k_f_* in the Cu region from 1.33 to 1.13 Å^−1^.

### 2.5 Electronic Origins of Oscillation Patterns

**Figure 5:**
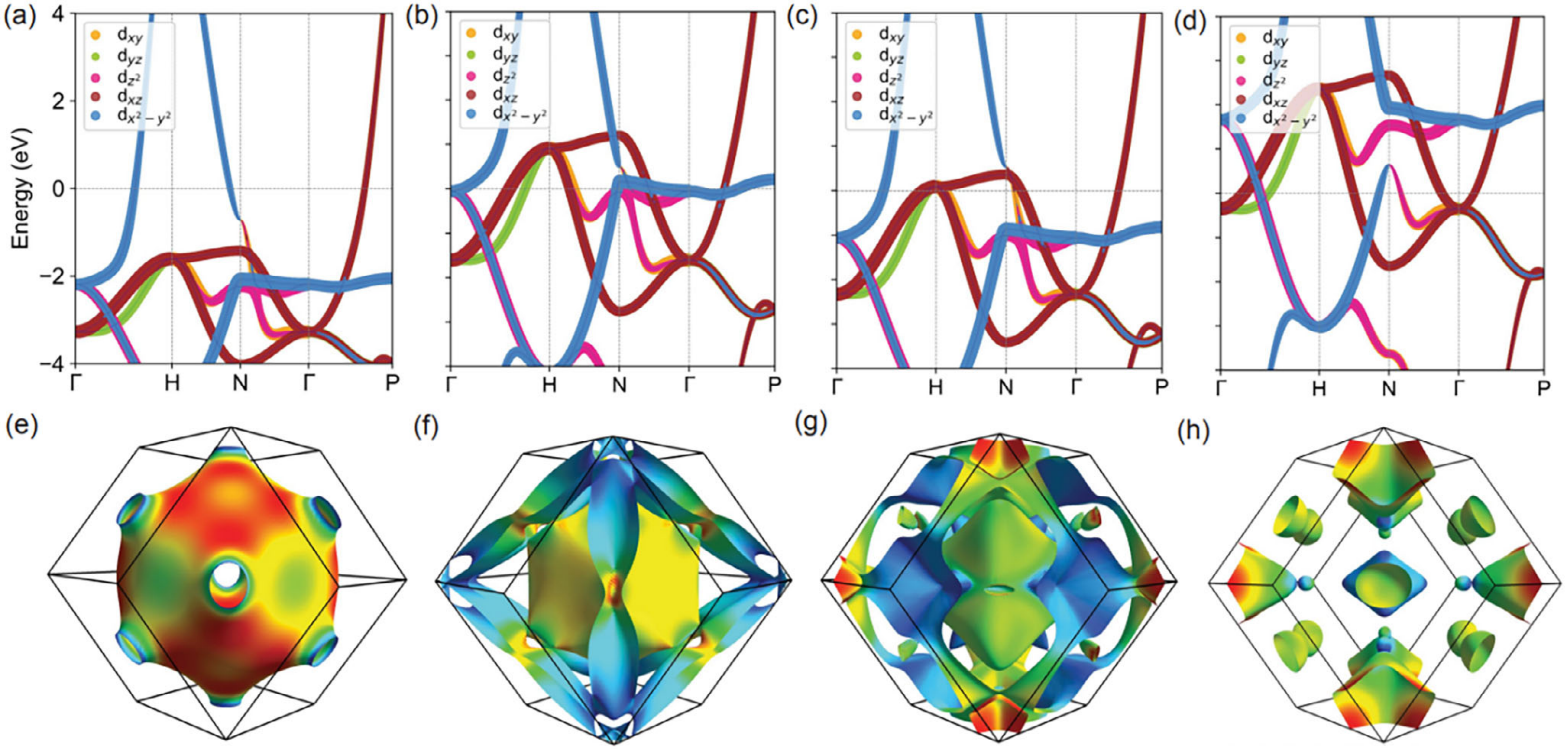
The band structure and Fermi surface of different systems. a) bcc Cu, b) nonmagnetic Fe, c) ferromagnetic Fe with majority spin, d) ferromagnetic Fe with minority spin, and e–h) their corresponding Fermi surface. The Fermi energy is set to zero.

As shown in Figure 3, the wavelength of Friedel oscillations is crucial in its impact on the interlayer binding. The Fermi wavevector, also known as the Fermi momentum, is a measure of the momentum of electrons at the Fermi level. The Brillouin zone of a bcc lattice is sketched in Figure S8 (Supporting Information). We have calculated the band structure of bcc‐Cu, nonmagnetic (NM) bcc‐Fe, and ferromagnetic (FM) bcc‐Fe, and plotted them in **Figure** **5**. H, N, and P represent the [001], [110], and [111] directions, respectively. The Fermi energy is set to zero. The Fermi wavevectors can be determined by the coordinates of points where the energy bands cross the Fermi surface.

We list the calculated Fermi wavevector *k_F_* and corresponding to the slope of bcc Fe, and of Cu along various directions in **Table** **2**. In describing the wavelength of Friedel oscillations, the *k_F_* to use depends on the specific direction of interest and the topology of the Fermi surface along that direction. The Friedel oscillation wavelength is inversely proportional to the magnitude of *k_F_*:

(9) $$\lambda =\frac{2\pi }{2k_{F}}=\frac{\pi }{k_{F}}$$

**Table 2:** The calculated Fermi wavevector *k_F_* (1/Å) and corresponding Fermi velocity (Ev × Å/ℏ) of Fe and Cu in bcc structure, along [001] directions. If there is more than one *k_F_*, the one corresponding to the energy band contributing most to the DOS is highlighted in bold.

	bcc Fe – FM				bcc Fe – NM		bcc Cu	
	Minority		Majority					
	*k_F_*	*v_F_* (*k*)	*k_F_*	*v_F_* (*k*)	*k_F_*	*v_F_* (*k*)	*k_F_*	*v_F_* (*k*)
Γ‐H (001)	0.45	1.08	1.00	3.20	0.07	0.26	1.33	7.69
	0.89	2.93	1.91	0.66	1.29	1.67		
	1.01	1.21	2.01	1.40	1.64	2.42		

In a material with multiple *k_F_* values along a specific direction due to multiple bands crossing the Fermi level, each *k_F_* contributes its own oscillatory component. The total Friedel oscillations will then be a superposition of oscillations with wavelengths derived from all contributing *k*_***F***_. Typically, a more significant contribution comes from the bands with a higher density of states at the Fermi level, as these states are more responsible for the scattering processes. The contribution of the *n*‐th energy band intersecting the Fermi level along a particular path to the density of states (DOS) at the Fermi level can be computed as follows:

(10) $$D_{n}\;\left(E_{F}\right)=\int_{BZ}\delta \left(E_{F}-E_{n}\left(k_{F}\right)\right)\;\frac{dk}{{\left(2\pi \right)}^{3}}$$

where δ(*E*_***F***_ − *E_n_* (*k*)) ensures that only states at the Fermi level are considered, and the integral is performed over the entire Brillouin zone (BZ). In three dimensions, if the band near the Fermi surface follows a simple dispersion relation *E_n_* (*k*), its contribution to the density of states is given by:

(11) $$D_{n}\;\left(E_{F}\right)=\sum_{k_{F}}\frac{1}{{\left(2\pi \right)}^{3}}\frac{dS}{\left|\nabla_{k}E\left(k\right)\right|}$$

where *k_F_* ​ represents the points where the band crosses the Fermi level, *dS* is an infinitesimal area element on the Fermi surface, and |∇_*k*_ *E*(*k*)| is the magnitude of the Fermi velocity:

(12) $$v_{F}\;\left(k\right)=\frac{1}{\hbar }\;\left|\nabla_{k}E\left(k\right)\right|$$

Therefore, the contribution of the *n*‐th energy band at *k_F_* to the DOS is in proportion to the inverse of the Fermi velocity at that point:

(13) $$D_{n}\left(E_{F},k\right)\propto \frac{1}{v_{F}\left(k\right)}$$

We find that in all three directions, spin polarization increases the value of *k_F_*. That means magnetization will reduce the wavelength of Friedel oscillations. Along the [001] direction, *k_F_* of NM‐Fe and Cu is smaller than that of FM‐Fe. This agrees with the observation in Figure 4. In addition, the *k_F_* values highlighted in bold in Table 2 are also in line with fitting parameters of Friedel oscillations in the atomic‐layer‐integrated charge density, indicating the interlayer binding is strongly correlated with both charge and spin Friedel oscillations. Our electronic structure analysis thus explains why, in Figure 3, the interlayer binding energy curve at the sharp interface is above the one at the diffuse interface.

### 2.6 Low‐Index (111) and (110) Fe/Cu Interfaces

For copper precipitates, in addition to the (001) interface, other low‐index interfaces such as (111) and (110) interfaces with comparable formation energy,^[^ ^28^ ^]^ also exist, and segregation of alloy atoms at these interfaces has been recently studied.^[^ ^29^ ^]^ The nearest interatomic distance is $\sqrt{2}a$ and $\sqrt{3}/2a$, in the (111) and (110) plane, respectively. Since the repulsive Fe/Cu interaction is the weakest in the (111) plane, we expect the 2D alloying will possibly occur in the atomic layer at the (111) interface.

Similarly, the interface structures for the (111) Fe/Cu interface have been investigated, and the results are displayed in Figure S9 (Supporting Information). Among all Cu concentrations in these three layers, the most stable Fe/Cu (111) interface can take three types of configurations, i.e., sharp, single, or double‐layer diffused, indicating a well‐defined boundary between pure Fe and Cu (111) layers. Simultaneous comparison of different interface configuration energies reveals that the double‐layer diffuse interface ($\Omega_{3}$) has the lowest total energy, while the sharp interface ($\Omega_{0}$), the single‐layer diffuse interfaces $\Omega_{2}$ and $\Omega_{3}$ are 1, 9, and 8 *m*eV per interfacial atom higher, respectively (Figure S9d, Supporting Information). Since the repulsion between Fe and Cu atoms in one layer is already very strong for the (110) plane, we only have explored Fe‐Cu mixing within a single layer. Our DFT calculations declare that the sharp interface, $\Omega_{0}$, has the lowest energy, and the diffuse interface configurations, $\Omega_{1}\;∼\Omega_{4}$ are at least 0.070 eV per atom higher in energy (Figure S10, Supporting Information).

To assess the role of entropy in stabilizing diffuse interfaces, we considered configurational, electronic, and vibrational contributions. For the (110) and (001) interfaces, the energy differences between sharp and diffuse configurations are sufficiently large that entropy effects can be neglected. In contrast, the (111) interface shows a smaller energy difference, and thus, entropy may play a more significant role. At 300 K, the total entropy difference was evaluated using

(14) $$\Delta S=S_{dif}\;-\;S_{shp}$$

where, *S_dif_* and *S_shp_* denote the entropy of the diffuse and sharp interfaces, respectively. The electronic and vibrational entropy contributions at 300 K were found to be −0.0075 and +1.8 meV per atom, respectively. The configurational part of the entropy is calculated here.

(15) $$S=-k\sum_{i=1}^{n}x_{i}\ln\left(x_{i}\right)$$

For $\Omega_{1}$, *S*≅0.562*k*/*atom*, and for $\Omega_{2}$, *S*  =  0.693*k*/*atom*. At *T*  =  300K, the *S* × *T* term is ≈+0.014 and +0.018 eV per atom, indicating that configurational entropy dominates. These results were further incorporated into the evaluation of Fe–Cu interfacial composition as a function of temperature.

Although the 2D alloying in the (111) case can be well understood by the entropy contribution, we have still calculated the interlayer binding energy near the Fe/Cu (111) interface (Figure S11, Supporting Information). It is found that the sharp and diffuse curves at the interface are coincident with each other much better than in the (001) case, both with and without spin polarization. Oscillations are visible in the case of a sharp interface, though with a much lesser magnitude than in the (001) case. The magnitude of the Friedel oscillations is determined not only by the Fermi surface geometry, but also by the strength of the disturbance introduced by the interface, and temperature as well. Quantitative analyses of the magnitude of the Friedel oscillations will be a key task in our future work.

Finally, we want to address the effect of temperature. We have calculated the formation energies of (001), (111), and (110) Fe/Cu interfaces to be 38, 33, and 31 meV Å^−^^2^, in good agreement with previous reports.^[^ ^28^ ^]^ Calculations based on Gibbs–Wulff construction rules^[^ ^30^ ^]^ indicate that the diffuse (001) and (111) facets dominate the interfacial area, contributing approximately 64%. On the (111) facet, the small energy difference (∼1 meV per atom) between diffuse and sharp interfaces renders noticeable entropy contributions. With the inclusion of entropy contribution, the predicted Fe content is about ∼32% at 0 K and ∼27% at 300 K (Figure S12, Supporting Information), in reasonable agreement with the Atom Probe Tomography (APT) measurements (up to 50%) Fe,^[^ ^5^ ^]^ given the considerable experimental uncertainty. Detailed computational procedures are provided in the Supporting Information.

## 3 Effect of 2D Alloying on the Interfacial Segregation

We now scrutinize whether there is a remarkable difference in the segregation behavior of impurities and alloy elements at the sharp and single‐layer diffuse Fe/Cu (001) interfaces. We have studied four kinds of interstitial atoms, hydrogen (H), helium (He), boron (B), and carbon (C), and four types of substitutional atoms, aluminum (Al), chromium (Cr), manganese (Mn), and nickel (Ni). The supercells are the same as those depicted in Figure 2. Due to the directional bonding, the most stable interstitial site of H and He in bulk Fe and Cu is the tetrahedral interstitial site (TIS), while C and B prefer to octahedral interstitial site (OIS), which is consistent with the previous calculations.^[^ ^31^, ^32^, ^33^ ^]^ The positions of H/He and C/B atoms in the vicinity of Fe/Cu (001) interfaces, both sharp and single‐layer diffuse types, are shown in Figure S13 (Supporting Information). The segregation energy is defined as the energy lowering when an impurity or alloying element moves from deep inside Fe to the most stable position at the interface. Thus, a negative value means this element will segregate to the interface.

**Figure 6:**
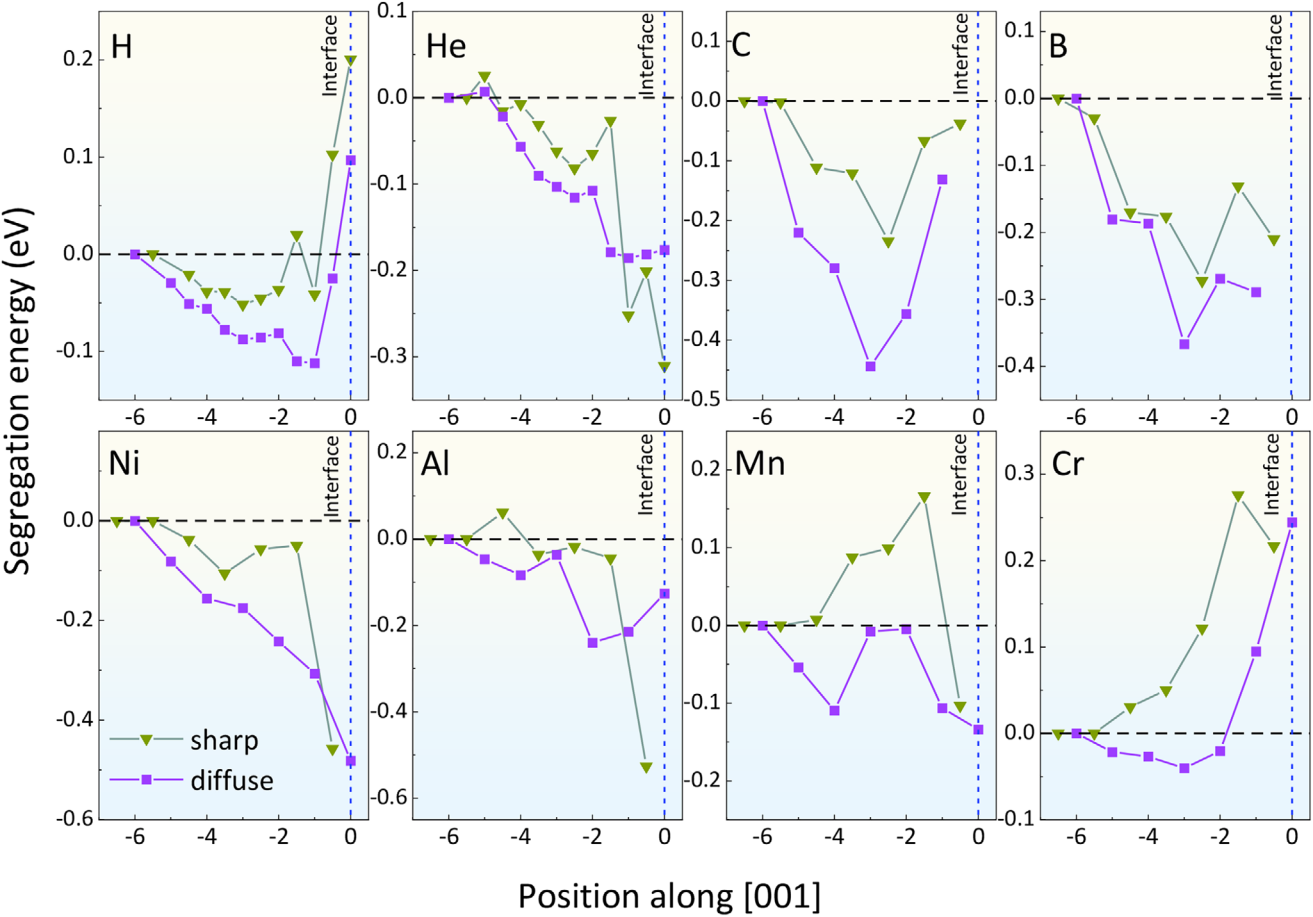
Effect of 2D alloying on the interfacial segregation. Segregation energy of interstitial (H, He, C, and B) and substitutional atoms (Al, Cr, Mn, and Ni) at the sharp (green triangles) and single‐layer diffuse (purple squares) (001) interfaces. Note that interfaces are positioned at zero.

The calculated segregation energies of all these eight elements at the sharp (green triangles) and single‐layer diffuse (purple squares) (001) interfaces are shown in **Figure** **6**. We see that the interstitial H prefers the boundary to the Fe matrix for all types of interfaces. However, the strength of segregation differs, and it is −0.04 and −0.11 eV for the sharp and the diffuse situations, respectively. This means 2D alloying at the Fe/Cu interfaces enhances their capability of capturing H.^[^ ^4^ ^]^ On the contrary, the segregation tendency of He is stronger at the sharp (−0.31 eV) than at the diffuse interface (−0.19 eV). Like for H, the assumption of a sharp interface will underestimate the segregation tendency of C and B, and the underestimation induced by assuming a sharp interface is −0.21 eV for C, and −0.10 eV for B.

As for the substitutional elements, there is experimental evidence that Ni, Al, and Mn are enriched at the boundary of Cu precipitates,^[^ ^34^, ^35^ ^]^ with the segregation of Ni being stronger than Mn and Al.^[^ ^36^ ^]^ Our DFT calculations show thermodynamic driving force for segregation of Ni, Al, and Mn at both sharp and diffuse interfaces along [001] orientations. At the Fe/Cu (001) interface, the segregation at the diffuse interface is slightly stronger than that at sharp interfaces for Ni and Mn, while it has the opposite trend for Al. Along [001] directions, the most striking influence of the interfacial 2D alloying is on Cr segregation. At the Fe/Cu (001) interface, Cr will have a slight segregation driving force (−0.04 eV) at the diffuse interface, while it will not segregate at the sharp interface.

In summary, except for Al and He, the assumption of sharp Fe/Cu (001) interfaces underestimates the segregation tendency for transition metal elements Cr, Mn, and Ni, and interstitial elements H, C, and B. Such an underestimation has important consequences on the efficiency of the strengthening effect of the Cu precipitates. Especially for H, 2D alloying makes (001) Fe/Cu interfaces as traps for H, thereby mitigating the formation of H bubbles.

## 4 Conclusion

To address the notable discrepancy between the low solubility of Fe in bulk Cu and the high Fe content in Cu precipitates, we have performed first‐principles DFT calculations on the energetics of the Fe/Cu interfaces with varying interfacial configurations. Our calculations reveal an unexpected characteristic of the interface between Cu precipitates and the Fe matrix. Contrary to the conventional views of an immiscibly sharp or a commonly miscible interface over several atomic layers, we have uncovered unique single‐layer (001) and double‐layer (111) Fe/Cu diffuse interfaces. Electronic structure analyses indicate that it is the magnetic Friedel oscillations that drive the formation of a single‐layer diffuse (001) interface. However, the magnetic Friedel oscillations are found to be the key factor in the (001) case. The Fermi wavevector, which determines the wavelength of Friedel oscillations, is crucial in its impact on the interlayer binding. The configurational entropy is found to drive the 2D alloying at the (111) interface. The 2D alloying at the Fe/Cu interfaces leads to a significant content of Fe in Cu precipitates due to their small size. Our DFT calculations on the segregation of interstitial atoms H, He, B, and C, and substitutional atoms Al, Cr, Mn, and Ni at the (001) interfaces provide strong evidence that the 2D alloying has a considerable effect on their segregation behavior. Especially, it introduces a H‐trapping effect at the interfaces, which is beneficial to improve the steel's resistance to H embrittlement. Our finding is a strong demonstration that quantum mechanical effects, such as Friedel oscillations, could have remarkable consequences also in structural materials such as steels.

## 5 Experimental Section

The calculations were performed using density functional theory (DFT).^[^ ^37^, ^38^ ^]^ via the Vienna ab initio Simulation Package (VASP).^[^ ^39^ ^]^ The exchange–correlation energy was treated using the generalized gradient approximation (GGA) with the PBE functional,^[^ ^40^, ^41^ ^]^ which has been validated in previous first‐principles studies of Cu precipitation in bcc Fe.^[^ ^28^ ^]^ Although meta‐GGA functionals such as SCAN may offer improved accuracy in certain magnetic systems, PBE has been shown to reliably reproduce interfacial energies and precipitate morphologies in Fe–Cu alloys while maintaining computational efficiency. A plane wave cutoff energy of 400 eV was used, achieving energy convergence within 10^−6^ eV per atom. Reciprocal space integrations were sampled with a Gamma‐centered Monkhorst‐Pack *k*‐mesh^[^ ^42^ ^]^ of 0.03Å^−1^. The details of the supercells were described in the following section. To verify whether the thickness of the slab model was sufficient, the 2 × 2 × 12 and 2 × 2 × 15 Fe/Cu (001) supercells were compared. The differences in optimized length of *a* and *c* axes were only 0.06% and 0.22%, respectively, indicating the sufficiency of the 2 × 2 × 12 supercell. The thicknesses of the supercells used to model the (110) and (111) interfaces were comparable to that of the (001) orientation. Finally, the VASPKIT program was used.^[^ ^43^, ^44^ ^]^ to post‐process the calculated data obtained by using the VASP code. Magnetic coupling parameters (J) were calculated using OpenMX in conjunction with the TB2J code. For OpenMX, a cutoff energy of 600 Ry and an energy convergence criterion of 10^−^⁶ Hartree were applied, with a 3 × 3 × 1 k‐point mesh and the GGA‐PBE exchange–correlation functional. The basis sets were chosen as Fe6.0H‐s3p2d1 and Cu6.0H‐s3p2d1. For TB2J, a 2 × 2 × 1 supercell was constructed to obtain the nearest‐neighbor J values.

## Conflict of Interest

The authors declare no conflict of interest.

## Supporting information



Supporting Information

## Data Availability

The data that support the findings of this study are available in the supplementary material of this article.
