# Supplementary material for: Two‐Dimensional Alloying at the Iron‐Copper Interface in Steel Driven by Magnetic Friedel Oscillations
Source: Adv Sci (Weinh). 2025 Oct 30;13(3):e14699. doi: 10.1002/advs.202514699 (PMC12806526; doi:10.1002/advs.202514699)
Supplement: Supplementary file 1 — Supporting Information [file ADVS-13-e14699-s001.docx]

Supporting Information

Two-Dimensional Alloying at Iron-Copper Interface in Steel Driven by Magnetic Friedel Oscillations

Wen-Qiang Xie^‡^, Jin-Li Cao^‡^, Jian-long Kou, Wen-Tong Geng^*^

W. Q. Xie, J. L. Kou, W. T. Geng

Department of Physics, Zhejiang Normal University, Jinhua 321004, China
E-mail: wtgeng@zjnu.edu.cn

W. Q. Xie
School of Materials Science and Engineering, Hainan University, Haikou 570228, China

J. L. Cao

Institute of Reactor Engineering and Technology, China Institute of Atomic Energy, Beijing 102413, China


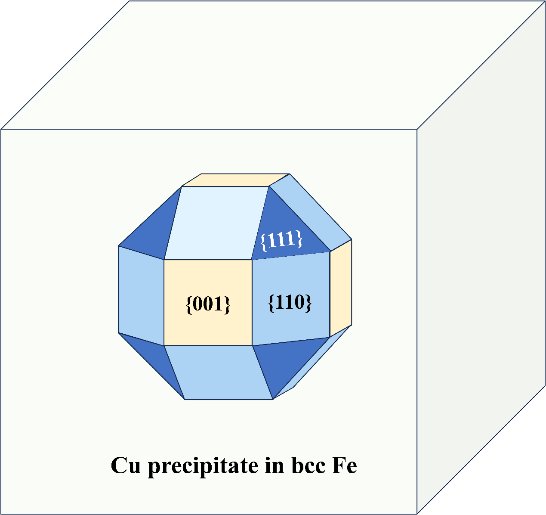


**Figure S1**. Illustration of Cu precipitates in bcc Fe.


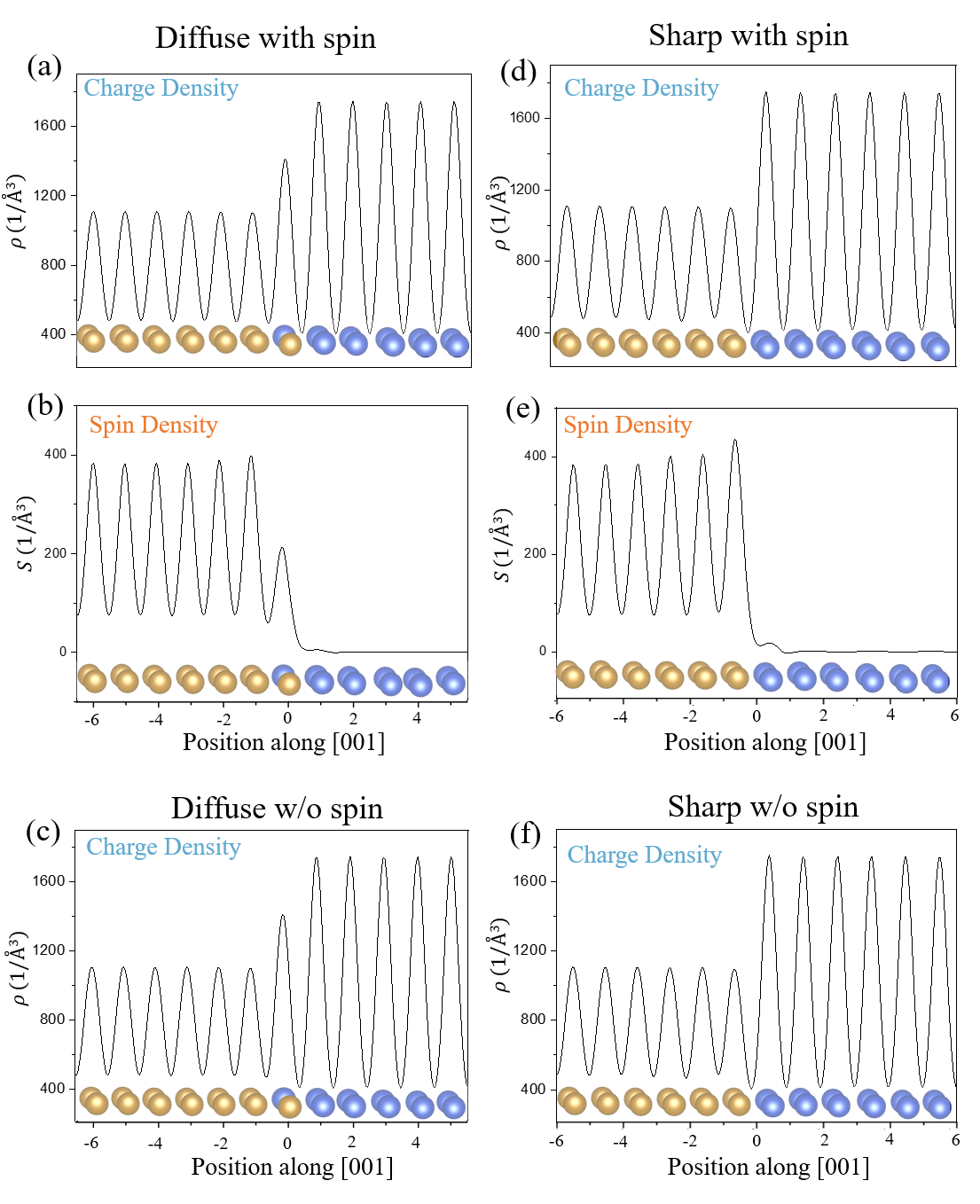


**Figure S2.** The electron density and spin density at (001) interfaces. (a-b) The (001) plane-averaged electron density, ρ, and spin density, *S*, near the diffuse (001) interface with spin-polarization. (c) ρ near the diffuse (001) without spin-polarization. (d-f) Same as (a-c), but for the sharp interface.

**
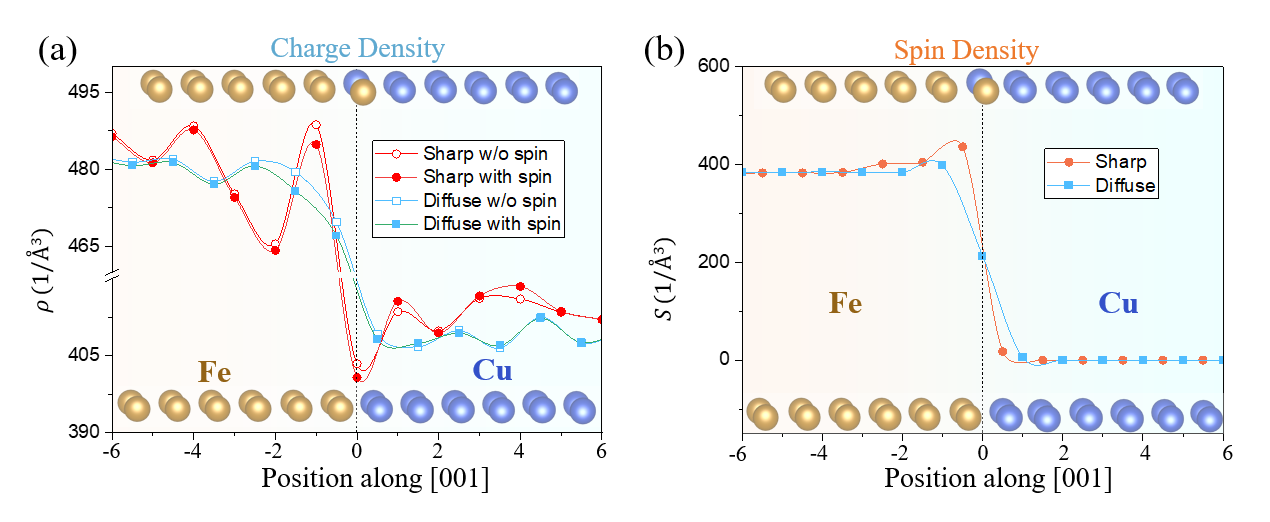
**

**Figure S3.** The electron density and spin density near (001) interfaces. (a) The minimum value of (001)-atomic-layer-averaged electron density, and (b) the maximum of spin density near the interface as illustrated in Fig. S2.


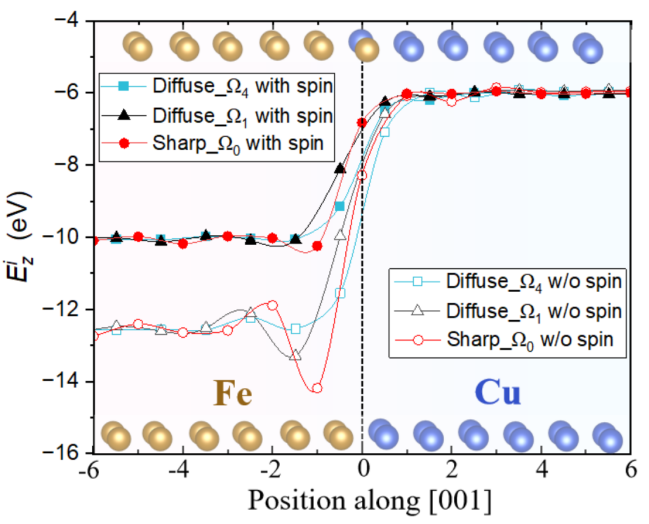


**Figure S4.** Interlayer binding energy (eV/interface) near the $\Omega_{0}$, $\Omega_{1}$, and $\Omega_{4}$ Fe/Cu (001) interfaces. The atomic configurations of these interfaces are illustrated in Fig. 2. Note that $\Omega_{0}$ and $\Omega_{4}$ correspond to the sharp and single-layer diffuse interfaces, respectively, discussed in Figure 3.


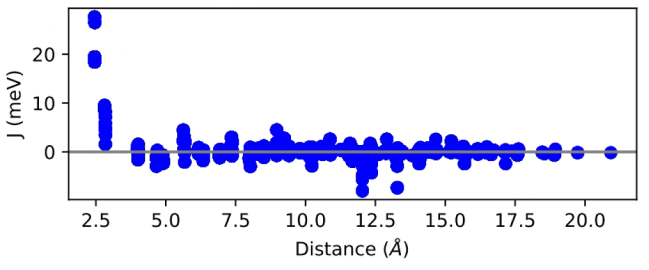


**Figure S5.** The isotropic magnetic interaction across the sharp (001) interface. Strong magnetic coupling is found to be primarily confined to a distance of 5 Å.


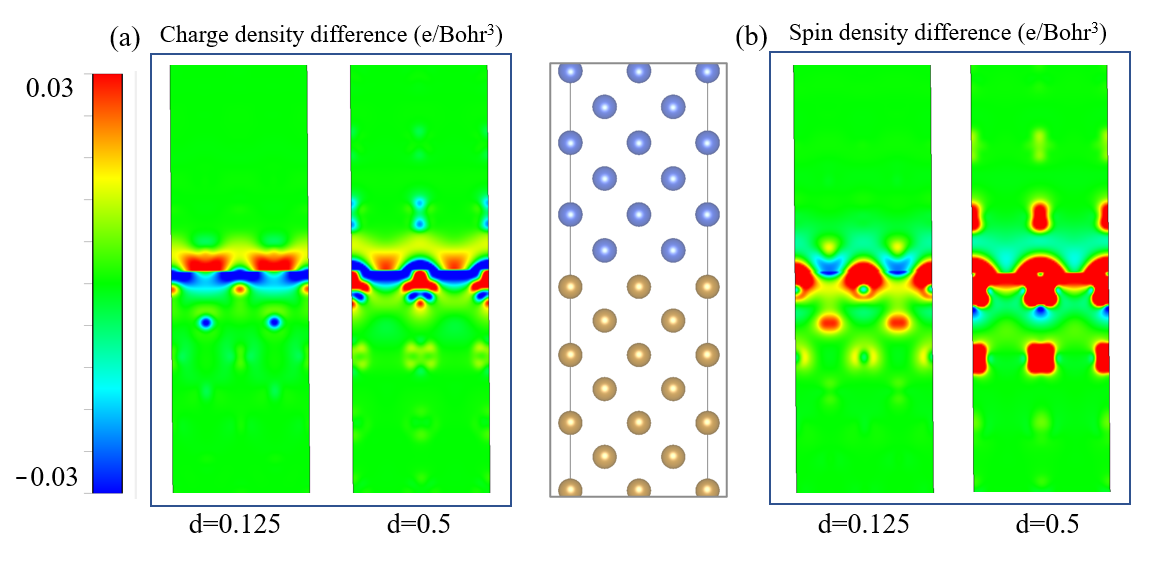


**Figure S6**. (a) Charge density difference and (b) spin density difference along (001) direction with various distance from the origin (d=0.125/0.5).


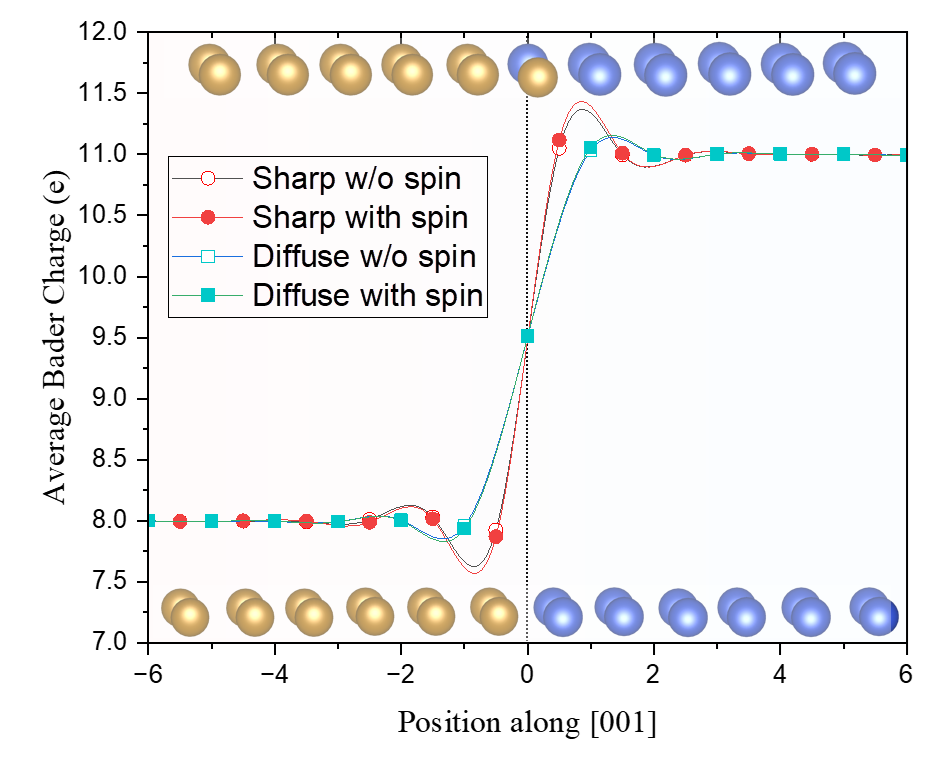


**Figure S7**. Layer-resolvered average Bader charge.


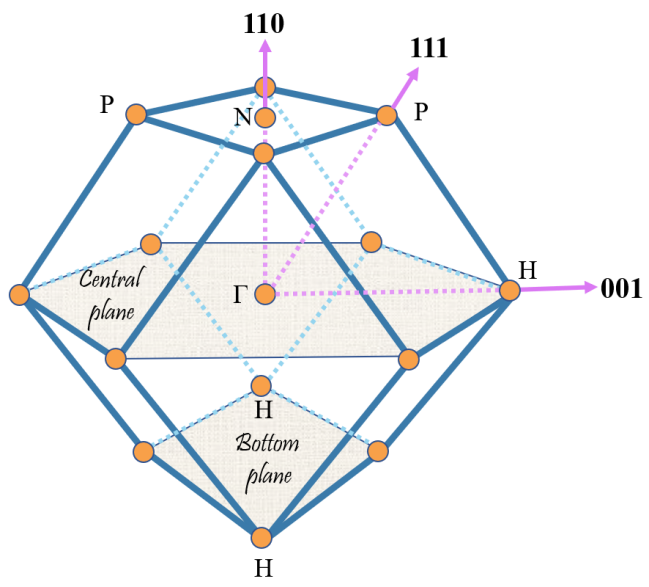


**Figure S8.** The Brillouin zone of bcc crystals.

**Figure S9.** Configurations and energies at the Fe/Cu (111) interface. (a) Supercell used model the (111) interface between Fe (brown) and Cu (blue). Three-layer grey atoms at one interface represent undesignated species, either Cu or Fe. (b) The relative energy of each configuration in reference to the most stable one. (c) The most stable configuration for each Cu concentration. (d) The relative energy (*m*eV per interfacial atom) of each possible configuration ($\Omega_{i}$) in reference to that of the sharp interface $\Omega_{0}$.


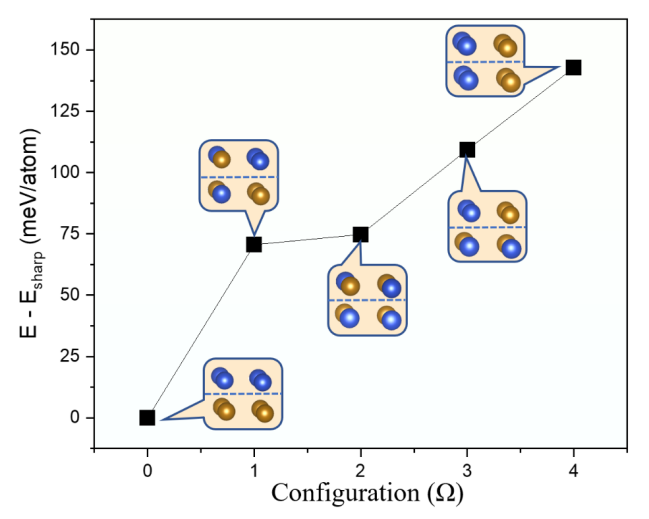


**Figure S10.** Configurations and energies at the Fe/Cu (110) interface. The relative energy (*m*eV per interfacial atom) of each possible configuration ($\Omega_{i}$) in reference to that of the sharp interface $\Omega_{0}$ along (110) interface.


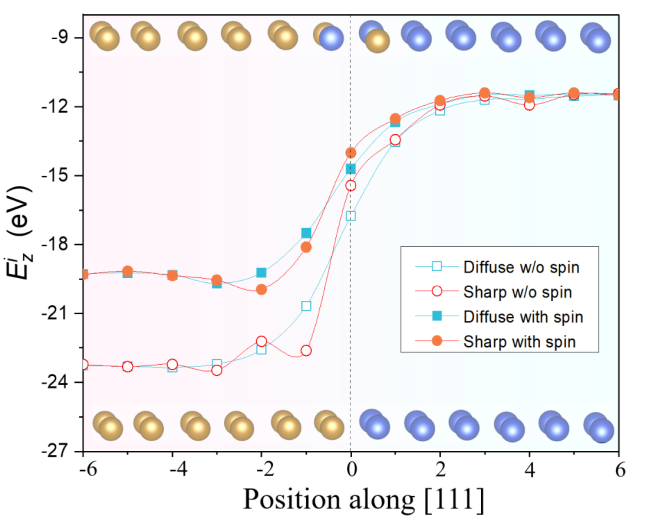


**Figure S11.** The interlayer binding energy (eV/interface) near the Fe/Cu (111) sharp and diffuse interface, with and without spin-polarization.

**
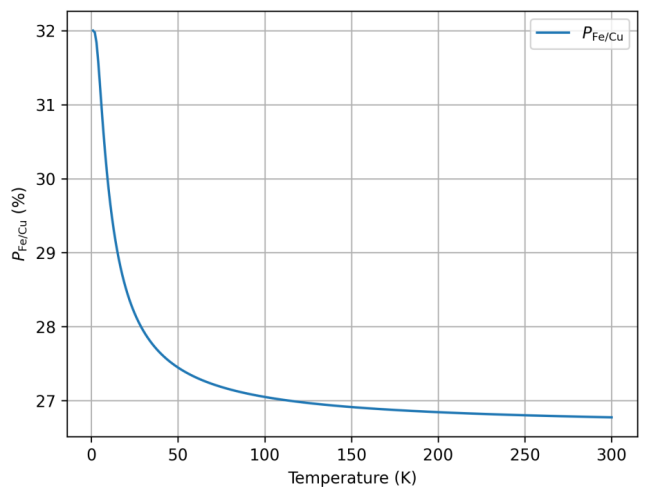
**

**Figure S12.** Fe content in Cu precipitates as a function of temperature. The calculation method can be found in the end of supplymentary.

**Figure S13.** Configurations of element segregation at the Fe/Cu (001) interface. Positions of interstitial atoms (H, He, C and B) in the vicinity of the sharp (a-b) and single-layer diffuse (c-d) Fe/Cu (001) interfaces. (a, c) H and He atoms occupy tetrahedral interstitial sites, (b, d) C or B atoms sit in octahedral interstitial sites. In the main text, both interstitial and substitutional positions are labelled by its distance in scale of interlayer spacing from the interface, denoted by dashed line.

**The calculation of Fe content in Cu precipitates as a function of temperature (Figure S12)**

Our calculation begin with the formation energies of the Fe/Cu interface, which is calculated as:

$E_{f}=\frac{E_{\mathrm{FeCu}}-E_{\mathrm{Cu}}-E_{\mathrm{Fe}}}{A}$ (S1)

Formation energies for the (001), (111), and (110) facets are 0.31, 0.46, and 0.18 eV/atom, respectively, corresponding to 38, 33, and 31 meV/Å², in good agreement with previous report.^[S1]^ Using Wulff’s theorem, the equilibrium crystal shape can be estimated via the Gibbs–Wulff construction, where the perpendicular distance $h_{i}$of each facet from the crystal center is proportional to its surface energy:^[S2]^

$\frac{h_{i}}{E_{f}}=C$ (S2)

Here, $h_{i}$ is the distance from the facet to the center, and $C$ is a constant. While exact facet areas require detailed geometric construction, a rough estimate assumes that the area is inversely proportional to the surface energy:^[S2]^

$P_{(001)}:P_{(111)}:P_{(110)}= \frac{1}{E_{f(001)}}:\frac{1}{E_{f(111)}}:\frac{1}{E_{f(110)}}=30:34:36$ (S3)

This indicates that the contributions from surface energies dominate over the energy differences among different Fe–Cu interface configurations. Accordingly, the diffuse interface primarily originates from the (001) and (111) facets, contributing ~64% of the total interface area.

For the (110) facet, the energy difference between the sharp and stable diffuse interfaces is ≥140 meV/atom, much larger than the combined electronic, vibrational, and configurational entropy contributions at 300 K. Therefore, the (110) facet remains primarily sharp, and its structure is insensitive to temperature. Similarly, the energy difference for the (001) facet is ≥40 meV/atom, again exceeding entropy contributions, so the interface is mainly diffuse, with minor temperature effects. As for the (111) facet, the energy difference between the double-layer diffuse and sharp interfaces is only ~1 meV/atom, making entropy contributions significant.

The fraction of diffuse interfaces ($P_{diff}$) can be estimated as:

$P_{diff}=P_{(111)}\times\frac{e^{-\frac{\Delta E_{diff(111)}}{KT}}}{1+e^{-\frac{\Delta E_{diff(111)}}{KT}}}+P_{(001)}$ (S4)

with

$\Delta E_{diff(111)}= \Delta E_{0}-T\Delta S$ (S5)

and

$\Delta S={\Delta S}_{conf}+{\Delta S}_{vib}+{\Delta S}_{ele}$ (S6)

and the corresponding Fe/Cu content ($P_{Fe/Cu}$) can be estimated as:

$P_{Fe/Cu}=P_{diff}\times50\%$ (S7)

Here, $\Delta E_{0}$ is the energy difference between double-layer diffuse and sharp interfaces on the (111) facet, ${\Delta S}_{conf}$, ${\Delta S}_{vib}$, and ${\Delta S}_{ele}$ denote configurational, vibrational, and electronic entropy contributions, respectively. Using this approach, the predicted Fe content is ~32% at 0 K and decreases to ~27% at 300 K. as illustrated in **Figure S12**. Our calculated Fe content agrees reasonably well with APT measurements (up to 50%), given the considerable experimental uncertainty.^[S3]^

**Reference**

S1. J. Wang, et al., “First-principles study on the equilibrium shape of nanometer-sized body-centered cubic Cu precipitates in ferritic steels,” *Comput. Materi. Sci.* 172 (2020): 109351.

S2. T. L. Einstein, Handbook of Crystal Growth: Equilibrium Shape of Crystals, 2nd ed., British Library (Amsterdam: Elsevier, 2015), ISBN 9780444563699.

S3. M. Fine, J. Liu, M. Asta, “An unsolved mystery: The composition of bcc Cu alloy precipitates in bcc Fe and steels,” *Mater. Sci. Eng. A.* 463 (2007): 271-274.
